# The burden of maternal morbidity and mortality attributable to hypertensive disorders in pregnancy: a prospective cohort study from Uganda

**DOI:** 10.1186/s12884-016-1001-1

**Published:** 2016-08-04

**Authors:** Annettee Nakimuli, Sarah Nakubulwa, Othman Kakaire, Michael Odongo Osinde, Scovia Nalugo Mbalinda, Nelson Kakande, Rose Chalo Nabirye, Dan Kabonge Kaye

**Affiliations:** 1 Department of Obstetrics and Gynecology, School of Medicine, College of Health Sciences, Makerere University, P.O. Box 7072, Kampala, Uganda; 2 Department of Obstetrics and Gynecology, Jinja Regional Hospital, Jinja, Uganda; 3 Department of Nursing, School of Health Sciences, College of Health Sciences, Makerere University, P.O. Box 7072, Kampala, Uganda; 4 Clinical, Operations and Health Services Research Program, Joint Clinical Research Centre, P. O. Box 10005, Kampala, Uganda

## Abstract

**Background:**

Hypertensive disorders of pregnancy are a major cause of morbidity and mortality. The objective was to estimate the disease burden attributable to hypertensive disorders of pregnancy in two referral hospitals in Uganda.

**Methods:**

Through a prospective cohort study conducted in Jinja and Mulago hospitals in Uganda from March 1, 2013 and February 28, 2014, hypertension-related cases were analyzed. Maternal near miss cases were defined according to the WHO criteria. Maternal deaths were also analyzed. The maternal near miss incidence ratio, the case-specific severe maternal outcome ratio, the case-specific maternal mortality ratio and the case-fatality ratio were computed.

**Results:**

Of 403 women with hypertensive disorders of pregnancy, 218 (54.1 %) had severe preeclampsia, 172 (42.7 %) had eclampsia, and 13 had chronic hypertension or Hemolysis, elevated liver enzymes or low platelets (HELLP) syndrome. The case-specific *maternal near miss incidence ratios* was 8.60 per 1,000 live births for all hypertensive disorders, 3.06 per 1,000 live births for severe preeclampsia and 5.11 per 1,000 live births for eclampsia. The case-specific *severe maternal outcome ratio* was 9.37 per 1,000 live births for all hypertensive disorders, and was 3.25 per 1,000 live births for severe preeclampsia and 5.61 per 1,000 live births for eclampsia. The case-specific *maternal mortality ratio* was 780 per 100,000 live births for all hypertensive disorders, and was 1940 per 100,000 live births for severe preeclampsia and 501 per 100,000 live births for eclampsia. The *case-fatality ratio* was 5.1 % overall (for all hypertensive disorders), but was 8 times higher for eclampsia compared to severe preeclampsia. Cyanosis, abnormal respiration, oliguria, circulatory collapse, coagulopathy, thrombocytopenia, and elevated serum lactate were significantly associated with severe maternal outcomes.

**Conclusion:**

There is high morbidity attributable to hypertensive disorders in pregnancy. Since some of the complications associated with morbidity can be recognized early, it is possible to prevent severe morbidity through early intervention with delivery, antihypertensive therapy and prophylactic magnesium sulphate treatment. The findings highlight the feasibility of implementing a facility-based surveillance system for severe maternal morbidity due to hypertensive disorders.

## Background

Hypertensive disorders in pregnancy are a major cause of morbidity and mortality among women and their offspring [1–4]. The burden of disease is a measure that assesses and compares the relative impact of different diseases or disorders on populations by quantifying health loss due to disease (or disorders) that remains after prevention, treatment, or rehabilitation efforts of the health system and society. For hypertensive disorders in pregnancy, the burden is not well documented in many countries worldwide, and few countries have national data on their incidence [5]. From systematic reviews, the crude incidence of eclampsia ranged from 0.1 to 8 % [5–7]. The case fatality ratio of eclampsia ranges from 0 to 1.8 % in high-income countries to 17.7 % in middle-income countries such as India, which reflects the gap in the quality of maternal health care [8–10]. Indeed, while no maternal death due to eclampsia occurred in a one-year period in Sweden [9], one hospital in India reported 11 eclampsia-related deaths [10].

There are several explanations for the high morbidity burden of severe preeclampsia and other hypertensive disorders in pregnancy. Preeclampsia is a disorder characterized by abnormal placentation with subsequent maternal inflammatory and vascular response. It manifests as a systemic inflammatory disease that may lead to multiple maternal organ damage in liver, kidneys, lungs, and central nervous system [11]. The placenta-related complications of the disorders include placental insufficiency, placental abruption, intrauterine growth restriction, preterm birth and intrauterine fetal death [11]. Related systemic complications of the disorders include thrombocytopenia, disseminated intravascular coagulation, acute pulmonary edema, cerebro-vascular disorders, and chronic hypertension, whose risk, in comparison to women without hypertension, increases by 3 to 25 times [12–14]. Local data from Uganda on the magnitude of the morbidity burden of hypertensive diseases was scanty. In particular, data where the WHO criteria of organ-system dysfunction [15] have been used to indicate severity of obstetric complications is not available. Yet these criteria have been validated. The objective was to assess the burden of severe maternal outcomes attributable to hypertensive disorders in pregnancy.

## Methods

### Study setting and design

This was a prospective cohort study of women admitted with pregnancy complications between March 1, 2013 and February 28, 2014. The study was conducted at Mulago and Jinja Hospitals. Mulago is Uganda’s national referral hospital and the teaching hospital for Makerere University. The hospital has more than 1500 beds, of which around 400 are maternity beds, and conducts over 30,000 deliveries per year. It has an 8-bed high dependency unit (HDU), a 6-bed severe preeclampsia unit and a 6-bed intensive care unit (ICU), where patients with severe forms of the hypertensive disorders are managed. Jinja is a large regional referral hospital that serves six district hospitals in Central and Eastern Uganda, with about 1000 beds of which around 200 are maternity beds and conducts more than 10,000 deliveries per year, but lacks intensive care facilities. All eligible patients with severe preeclampsia routinely receive magnesium sulfate for eclamptic seizure prophylaxis, in addition to parenteral antihypertensive therapy. Most patients referred with severe preeclampsia receive magnesium sulfate prior to transfer to the regional referral hospitals, as magnesium sulphate is one of the essential drugs available at district hospitals and peripheral health centres that provide basic emergency obstetric care. At the referral units, routine care for admitted patients with severe preeclampsia includes anticonvulsant and antihypertensive therapy, as well as monitoring of maternal well being (6- hourly pulse, blood pressure, urine output and general condition assessment), maternal investigations (for hepatic, hematological and renal dysfunction); and monitoring of fetal well being (uterine and umbilical artery Doppler in addition to 6-hourly fetal heart assessment).

### Data collection

The inclusion criteria was a diagnosis of severe preeclampsia (blood pressure of 160/100 mmHG or above, with albuminuria, with or without laboratory evidence of hematological dysfunction) . Women with the above diagnosis who consented to participate were recruited in the study, and were followed up to six months postpartum or death. Using an interviewer-administered questionnaire and through review of medical records, data was collected on socio-demographic characteristics, obstetric history, current pregnancy complications and pregnancy outcomes up to hospital discharge. All participants were then followed up to 12 weeks postpartum. Hypertensive disorders of pregnancy were classified into gestational hypertension, chronic hypertension, mild and severe preeclampsia and eclampsia according to the ACOG guidelines [1]. Data was collected on hematological, hepatic and renal function, in addition to biochemical investigations serum uric acid, lactate and electrolytes. The diagnosis of chronic hypertension was made from presence of hypertension in the first-trimester or a history of co-existing hypertension preceding the pregnancy. Patients with features of hepatic dysfunction, haemolysis or thrombocytopenia were classified as Hemolysis, Elevated liver enzymes, Low platelets (HELLP) syndrome if their presentation fitted the diagnostic criteria for HELLP syndrome.

Maternal near misses were classified using the WHO criteria for definition of maternal near miss [15]. Shock was defined as a reduction of 40 mm Hg of systolic Blood Pressure from baseline despite adequate fluid resuscitation, along with presence of perfusion abnormalities (such as oliguria and lactic acidosis) or presence of acute altered mental status. Acute respiratory distress syndrome (ARDS) was diagnosed according to the American-European Consensus Conference on ARDS [16]. Maternal mortality was considered as death occurring during pregnancy or within six weeks of delivery.

### Data analysis

Descriptive analysis was done whereby categorical variables are presented as frequencies and percentages while numerical variables are presented as means or medians (with standard deviations or inter-quartile ranges respectively). The following indicators for morbidity due hypertensive disorders were computed to highlight the burden due to the hypertensive disorders:

i) The case-specific *maternal near miss incidence ratios* due to hypertensive disorders was derived as the ratio of near miss per 1,000 live births respectively;
ii) The case-specific *severe maternal outcome ratio* due to hypertensive disorders as ratio of maternal death plus near misses per 1,000 live births;
iii) The *case-fatality ratio due to hypertensive disorders* determined as the proportion of deaths out of the total number of patients presenting with hypertensive disorders, expressed as a percentage;
iv) The case-specific *severe maternal mortality index* due to hypertensive disorders, derived as maternal deaths divided by (total deaths plus maternal near misses) expressed as a percentage;
v) The case-specific *maternal mortality ratio* due to hypertensive disorders expressed as all maternal deaths per 100,000 live births

Maternal complications and procedures used for patient management were compared among women who presented with the different clinical types of hypertensive disorders. The differences across the groups were assessed with Chi-square test and a p-value for trend reported.

### Ethical considerations

This research was part of a post-doctoral research project of the last author (DKK) entitled:

*“Evaluation and surveillance of the impact of maternal and neonatal near-miss morbidity on the health of mothers and infants in Jinja and Mulago hospitals”*. Ethical approval to conduct the study was obtained from the Ethics and research committees of Mulago hospital (REC 310–2012), the School of Medicine, Makerere University College of Health Sciences (REC 2012–172) and from Uganda National Council for Science and Technology. Permission to conduct the study was obtained from the department of Obstetrics and Gynaecology, Makerere University, and from Mulago National Referral Hospital and Jinja Hospital.

Participants gave written informed consent to be enrolled in the study and for their data to be included in the study. The languages used in the informed consent were English and the local languages (Luganda and Lusoga for Mulago and Jinja hospitals respectively). Participants included minors (aged 14-17years), as Uganda national guidelines for human subject research allow research on mature and emancipated minors in certain situations (such as in pregnancy), with prior approval of an institutional review board.

For those with very severe morbidity, consent was obtained retrospectively when they recovered, or consent was obtained from the next of kin to involve the patients’ in the study and/or to include the patients’ information in our data. Participants and their next of kin received assurances that participation was voluntary, and that participants were free to stop participation at any time without their decision affecting the care they were entitled to. All those with complications, and their newborns, were provided free medical care or, where necessary, were offered additional counseling or referred to get other support services not available at the two health facilities. Permission was obtained from the management of the two referral hospital (and from the study participants) to review the participants’ records.

## Results

Of the 3100 women with severe obstetric complications, 403 (13.0 %) had hypertensive disorders. Table 1 shows the characteristics of women with hypertensive disorders. The different groups of hypertensive disorders differed significantly across age group, parity category, and mode of delivery, but did not differ according to timing of the disorder (before or after hospital admission) or on referral status. The prevalence of caesarean section was 73.0 % overall, but was 13 % higher for severe preeclampsia compared to eclampsia. However, eclampsia cases had twice as high incidence of severe maternal outcomes (maternal death and near miss combined) or maternal death (compared to severe preeclampsia). Table 2 shows the maternal morbidity indicators associated with hypertensive disorders. HELLP syndrome had the highest case-specific *severe maternal mortality index* (maternal deaths divided by total maternal deaths plus maternal near misses) as a percentage.

**Table 1:** Characteristics of women with hypertensive disorders of pregnancy

Characteristics	All participants	Severe preeclampsia	Eclampsia	Chronic hypertension	HELLP syndrome	p-value
	*n* = 403 n(%)	*n* = 218 n(%)	*n* = 172 n(%)	*n* = 4 n(%)	*n* = 9 n(%)	
*Age category* (years)						<0.001
Age <19	45 (11.2)	7 (3.2)	37 (21.5)	0 (0.0)	1 (21.2)	
19–35	168 (41.7)	89 (40.8)	74 (43.0)	1 (25.0)	4 (44.4)	
<36	190 (47.1)	122 (56.0)	61 (35.5)	3 (75.0)	4 (44.4)	
*Parity*						0.017
Primipara	167 (41.4)	83 (38.1)	78 (45.3)	1 (25.0)	5 (55.5)	
Para2-4	176 (43.7)	99 (45.4)	74 (43.0)	0 (0.0)	3 (33.3)	
Parity 5 or higher	60 (14.9)	36 (16.5)	20 (11.7)	3 (75.0)	1 (11.2)	
*Referral status*						0.493
Yes	265 (65.8)	141 (64.7)	113 (65.7)	3 (75.0)	8 (88.9)	
No	138 (34.2)	77 (35.3)	59 (34.3)	1 (25.0)	1 (11.1)	
*ΩTiming of complications*						0.907
Occurred prior to admission	183 (45.4)	101 (46.3)	76 (44.2)	2 (50.0)	4 (44.4)	
Occurred prior to admission and new complications occurred after admission	137 (34.0)	71 (32.6)	60 (34.9)	2 (50.0)	4 (44.4)	
Occurred after admission	83 (20.6)	46 (21.1)	36 (20.9)	0 (0.0)	1 (22.2)	
*Mode of delivery*						0.001
Vaginal	90 (22.3)	33 (15.1)	51 (29.6)	1 (25.0)	5 (55.5)	
Caesarean delivery	294 (73.0)	175 (80.3)	113 (65.7)	3 (75.0)	4 (44.5)	
Vacuum extraction	19 (4.7)	14 (14.6)	5 (4.7)	0 (0.0)	0 (0.0)	
*Severe maternal outcomes*						0.001
Yes	162 (40.2)	84 (38.5)	144 (83.7)	4 (100.0)	9 (100.0)	
No	241 (59.8)	134 (61.5)	28 (16.3)	0 (0.0)	0 (0.0)	
*Maternal death*						0.009
Yes	20 (5.0)	7 (3.2)	13 (7.6)	0 (0.0)	2 (22.2)	
No	383 95.0)	211 (96.8)	157 (92.4)	4 (100.0)	7 (77.8)	

Ω Timing of complication in relation to admission

**Table 2:** Maternal morbidity and mortality indicators associated with hypertensive disorders of pregnancy

Indicator	Overall ratio	Case-specific ratio
The case-specific *maternal near miss incidence ratios* (ratio of near miss due to hypertensive disorders per 1,000 live births)	8.60	Severe preeclampsia 3.06
		Eclampsia 5.11
		Chronic hypertension 0.15
		HELLP syndrome 0.27
The case-specific *severe maternal outcome ratio*(ratio of maternal death plus near misses from hypertensive disorders per 1,000 live births)	9.37	Severe preeclampsia 3.25
		Eclampsia 5.61
		Chronic hypertension 0.15
		HELLP syndrome 0.35
The case-specific *maternal mortality ratio*(maternal deaths per 100,000 live births)	780	Severe preeclampsia 1940
		Eclampsia 503
		Chronic hypertension 0.0
		HELLP syndrome 770
The case-specific *severe maternal mortality index* (maternal deaths divided by total deaths plus maternal near misses) as a percentage	8.3	Severe preeclampsia 6.0
		Eclampsia 9.0
		HELLP syndrome 22.2
The *case-fatality rate for hypertensive disorders*(proportion of deaths out of the total number of patients presenting with specific complications) as a percentage.	5.1	Severe preeclampsia 2.3
		Eclampsia 17.8
		HELLP syndrome28.6

Table 3 shows the incidence of the different types of hypertensive disorders. Of these, 218 (54.1 %) were severe preeclampsia, while 172 (42.7 %) had eclampsia, and 13 included HELLP syndrome and chronic hypertension. Of the 403, 265 (65.8 %) were referrals: 141 (64.7 %) of the 218 with severe preeclampsia were referrals, while 113 (65.7 %) of the 172 women with eclampsia were referrals.

**Table 3:** Incidence of the hypertensive disorders of pregnancy in relation to maternal outcomes

Hypertensive disorder	All Hypertensive disorders	Maternal deaths	Maternal near miss	NLTC
	Number (percentage)	Number (percentage)	Number (percentage)	Number (percentage)
	*N* = 403	*N* = 20	*N* = 222	*N* = 161
Severe Preeclampsia	218 (54.1)	5 (25.0)	79 (11.4)	134 (83.20)
Eclampsia	172 (42.7)	13 (65.0)	132 (19.0)	27 (16.8)
Chronic Hypertension	4 (1.0)	0 (0.0)	4 (0.6)	0 (0.0)
HELLP Syndrome	9 (2.2)	2 (10.0)	7 (1.0)	0 (0.0)

*Abbreviation: NLTC* Non life-threatening obstetric complications

Table 4 shows the characteristics of the participants assessed according to the diagnostic criteria for definition of severe maternal outcomes (maternal near miss cases and maternal deaths. Cyanosis, abnormal respiration, oliguria, loss of consciousness, circulatory collapse or shock, coagulopathy, presence of thrombocytopenia, elevated serum lactate, the need for dialysis, and admission to the high dependency unit were significantly associated with severe maternal outcomes for eclampsia compared to severe preeclampsia.

**Table 4:** Diagnostic criteria used for the definition of severe maternal outcomes (maternal near miss cases or maternal deaths) in hypertensive disorders of pregnancy

Characteristics	All participants	Severe preeclampsia	Eclampsia	Chronic hypertension	HELLP syndrome	p-value
*Cyanosis*						<0.001
Present	169 (41.9)	50 (22.9)	112 (65.1)	2 (50.0)	5 (55.5)	
Absent	234 (58.1)	168 (77.1)	60 (34.9)	2 (50.0)	4 (44.4)	
*∞Abnormal respiration*						<0.001
Yes	186 (46.2)	61 (28.0)	115 (66.9)	3 (75.0)	7 (77.7)	
No	217 (53.8)	157 (72.0)	57 (33.1)	1 (25.0)	2 (22.2	
*Oliguria*						<0.001
Yes	234 (58.1)	168 (77.1)	63 (36.6)	1 (25.0)	2 (22.2)	
No	169 (41.9)	50 (22.9)	109 (63.4)	3 (75.0)	7 (77.7)	
*Loss of consciousness*						<0.001
Yes	110 (27.3)	31 (14.2)	75 (55.2)	0 (0.0)	4 (44.4)	
No	293 (72.7)	187 (85.8)	97 (44.8)	4 (100.0)	5 (55.5)	
*Shock*						<0.001
Yes	170 (42.2)	54 (24.7)	105 (61.0)	3 (75.0)	8 (88.8)	
No	233 (57.8)	162 (75.3)	67 (39.0)	1 (25.0)	1 (11.1)	
*Coagulopathy*						<0.001
Yes	136 (33.7)	36 (16.5)	91 (52.9)	2 (50.0)	7 (77.7)	
No	267 (66.3)	182 (83.5)	81 (47.1)	2 (50.0)	2 (22.2	
*ΩOxygen*						<0.001
Yes	182 (45.2)	61 (28.0)	111 (64.5)	2 (50.0)	9 (100.0)	
No	221 (54.8)	157 (72.0)	61 (35.5)	2 (50.0)	0 (0.0)	
*Thrombocytopenia*						<0.001
Yes	183 (45.4)	54 (24.7)	118 (68.6)	2 (50.0)	7 (77.7)	
No	220 (54.6)	162 (75.3)	54 (31.4)	2 (50.0)	2 (22.2)	
*Elevated Serum Creatinine*						<0.001
Yes	152 (37.7)	46 (21.1)	98 (57.0)	2 (50.0)	6 (66.7)	
No	251 (62.3)	172 (78.9)	74 (43.0)	2 (50.0)	3 (33.3)	
*Elevated serum Bilirubin*						<0.001
Yes	141 (35.0)	39 (17.9)	93 (54.1)	2 (50.0)	7 (77.7)	
No	262 (65.0)	179 (82.1)	79 (45.9)	2 (50.0)	2 (22.2	
*Elevated serum Lactate*						<0.001
Yes	136 (33.7)	34 (15.6)	95 (55.2)	2 (50.0)	5 (55.5)	
No	267 (66.3)	184 (84.4)	77 (44.8)	2 (50.0)	4 (44.4)	
*Use of Dialysis*						<0.001
Yes	33 (8.2)	7 (3.3)	23 (13.4)	1 (25.0)	2 (22.2)	
No	370 (91.8)	211 (96.7)	149 (86.6)	3 (75.0)	7 (77.7)	
Need for ICU or HDU admission						<0.001
Yes	208 (51.6)	65 (29.8)	132 (76.7)	3 (75.0)	8 (88.8)	
No	195 (48.4)	153 (70.2)	40 (23.3)	1 (25.0)	1 (11.1)	

∞Breathing rate more than 40 or less than 6 per minute; ΩOxygen saturation less than 90 % for more than 60 min; *ICU* Intensive care unit, *HDU* High dependency obstetric unit

## Discussion

Our study shows that hypertensive disorders of pregnancy constitute a high burden of morbidity and mortality as indicated by the morbidity and mortality indices. As noted in literature [17–20], the disorders include chronic (pre-existing) hypertension, gestational (transient) hypertension, preeclampsia/eclampsia, preeclampsia superimposed on chronic hypertension and eclampsia. Both gestational hypertension and preeclampsia/eclampsia may be associated in later life with hypertension and/or cardiovascular disease [17–20].

The burden of hypertensive disorders of pregnancy seems higher than that from the published literature from high income countries [1–4]. While the case fatality rate for eclampsia ranged from 0 to 1.8 % in high-income countries [8], it was 17.7 % in India [10] and was 2.3 in 2014 in a study from Uganda [21]. Such a wide range is due to both differences in incidence and quality of obstetric care for hypertensive disease in pregnancy. For our study, the high burden is associated with delayed recognition of the hypertensive disorders, delay in accessing vital services in peripheral units, delay in accessing prompt care, and poor quality of obstetric care in general [21–25]. In addition, the health services are constrained by limited supplies and equipment, poor skills and few numbers of lower-level healthcare providers to recognize and manage complications prior to referral, and high patient loads [21–25].

This high burden of hypertensive disorders in pregnancy is similar to that in other Eastern African countries [26–29] where hypertensive disorders are among the commonest causes of severe morbidity and even mortality. This calls for increased investments to increase research into how to address this burden in particular and on how to improve the quality of obstetric care in general, particularly improving clinical, laboratory and critical care facilities. This is necessary as diagnosis of maternal near miss using the organ/system dysfunction criteria requires efficient diagnostic and laboratory facilities. Yet, even the routine assessment of hepatic, renal and hematological function may not be available in many resource-limited settings where cases of severe preeclampsia/eclampsia and other obstetric disorders are managed [30]. In consideration of this difficulty in routinely conducting some of the vital biochemical laboratory investigations, the WHO criteria for definition of a near miss (in limited-resource settings such as in Uganda) may need to be revised to focus mainly on clinical and management-based criteria.

There are several explanations for the high burden of morbidity in our study. Many patients presented with multisystem involvement, as commonly occurs in severe hypertensive disorders [31–33], often leading to multiple organ failure and death. There was a high caesarean section rate in our study. The reason for the high caesarean section rate is partly due to the high incidence of eclampsia, presence of obstetric co-morbidities such as previous caesarean section (which rule out possibility of induction of labor or vaginal birth). In addition, many patients presented with severe preeclampsia remote from term, in which case caesarean section is the preferred mode of delivery. For instance, the incidence of caesarean section was 13 % higher for severe preeclampsia compared to eclampsia, due to more cases of prior caesarean deliveries in patients with severe preeclampsia. In addition, cases of severe preeclampsia had nearly half as many severe maternal outcomes (death or near miss) or maternal death (compared eclampsia), which indicates the high burden of co-morbidities in patients with hypertensive disorders.

Conceptua1lly, maternal near misses represent a point on the continuum where good health and death are the extreme points [31], where the complications could be described as uncomplicated, complicated (not life-threatening), severely complicated (life-threatening) or fatal [31–35], and where outcomes depend on both the severity of illness and the quality of care [35]. Our findings in Table 4 show that many patients with severe preeclampsia and eclampsia presented with organ/system dysfunction related to the hepatic, renal, neurological, pulmonary and coagulatory system dysfunction. In addition, patients with eclampsia were more likely to present with organ-system dysfunction compared to those with severe preeclampsia. The WHO criteria were validated by the WHO Working Group using markers of dysfunction and total maximum SOFA (sequential organ failure assessment) in obstetric patients [36, 37]. From our study, presence of cyanosis, abnormal respiration, oliguria, loss of consciousness, circulatory collapse or shock, coagulopathy, thrombocytopenia, elevated serum lactate, the need for dialysis, and admission to the high dependency unit were significantly may be interpreted as potential risk markers for severity of hypertensive disorders, as they occurred more commonly in cases of eclampsia [38–42]. They are also markers of or prognostic factors for organ dysfunction and organ failure [43–46]. Several studies have shown that the higher the SOFA score, the higher the mortality [43–46]. Future studies should validate the SOFA scoring system among patients with severe obstetric morbidity in our setting. In addition, studies should evaluate and validate risk prediction models for adverse outcomes and prognostic factors using morbidity indices and SOFA scores.

## Conclusion

Our study shows that hypertensive disorders cause a high burden of morbidity as indicated by the morbidity indices. This calls for interventions to reduce the burden and improve quality of care in hypertensive disorders of pregnancy. The findings highlight the feasibility of implementing a surveillance system for severe maternal morbidity from which data to inform interventions can be generated.

## Abbreviations

ACOG, American College of Obstetrics and Gynecology; AKI, Acute kidney injury; ARDS, Acute respiratory distress syndrome; CKD, Chronic kidney disease; HDU, High dependency obstetric unit; HELLP syndrome, Syndrome of hemolysis, elevated liver enzymes and low platelets; ICU, intensive care unit; REC, Research and Ethics Committee; SD, standard deviation; SOFA, sequential organ failure assessment; WHO, World Health Organization.
